# Efficacy and Safety of the PCSK9 Inhibitor Evolocumab in Patients with Mixed Hyperlipidemia

**DOI:** 10.1007/s10557-016-6666-1

**Published:** 2016-05-30

**Authors:** Robert S. Rosenson, Terry A. Jacobson, David Preiss, C. Stephen Djedjos, Ricardo Dent, Ian Bridges, Michael Miller

**Affiliations:** Mount Sinai Heart, Cardiometabolics Unit, Icahn School of Medicine at Mount Sinai, 1425 Madison Ave, MC1 Level, New York, NY 10029 USA; Emory University, 201 Dowman Drive, Atlanta, GA 30322 USA; Clinical Trial Service Unit and Epidemiological Studies Unit, Oxford University, Richard Doll Building, Old Road Campus, Roosevelt Drive, Oxford, OX3 7LF UK; Amgen Inc., One Amgen Center Dr, Thousand Oaks, CA 91320 USA; Amgen (Europe) GmbH, Dammstrasse 23, 6300 Zug, Switzerland; Amgen Ltd, 240 Cambridge Science Park, Milton, Cambridge, CB4 0WD UK; University of Maryland School of Medicine, 655 W Baltimore St, Baltimore, MD 21201 USA

**Keywords:** Apolipoprotein, High-density lipoprotein, Low-density lipoprotein-cholesterol, Proprotein convertase subtilisin/kexin type 9, Triglycerides

## Abstract

**Purpose:**

Evolocumab significantly reduces low-density lipoprotein-cholesterol (LDL-C); we investigated its effects on LDL-C lowering in patients with mixed hyperlipidemia.

**Methods:**

We compared the efficacy and safety of evolocumab in hypercholesterolemic patients selected from the phase 2 and 3 trials who had fasting triglyceride levels ≥1.7 mmol/L (150 mg/dL elevated triglycerides) and <1.7 mmol/L (without elevated triglycerides). Fasting triglyceride level ≥ 4.5 mmol/L at screening was an exclusion criterion for these studies, but post-enrollment triglyceride levels may have exceeded 4.5 mmol/L (400 mg/dL). Efficacy was evaluated in four phase 3 randomized studies (*n* = 1148) and safety from the phase 2 and 3 studies (*n* = 2246) and their open-label extension studies (*n* = 1698). Efficacy analyses were based on 12-week studies, while safety analyses included data from all available studies. Treatment differences were calculated vs. placebo and ezetimibe after pooling dose frequencies.

**Results:**

Mean treatment difference in percentage change from baseline in LDL-C for participants with elevated triglycerides and those without elevated triglycerides (mean of weeks 10 and 12) with evolocumab was approximately −67 % vs. placebo and −42 % vs. ezetimibe (all *P* < 0.001) compared to −6 % vs. placebo and −39 % vs. ezetimibe, respectively. Treatment differences for evolocumab vs. placebo and ezetimibe followed a similar pattern for non–high-density lipoprotein (HDL-C) and apolipoprotein B. Evolocumab was well tolerated, with balanced rates of adverse events leading to discontinuation of evolocumab vs. comparator (placebo and/or ezetimibe).

**Conclusion:**

The significant reductions of atherogenic lipids including LDL-C, non–HDL-C, and apolipoprotein B seen with evolocumab are similar in patients with and without mixed hyperlipidemia.

## Introduction

Evolocumab (AMG 145; Repatha®; Amgen Inc., Thousand Oaks, CA), a fully human immunoglobulin G2 monoclonal antibody, inhibits proprotein convertase subtilisin/kexin type 9 (PCSK9)–mediated proteolytic degradation of hepatic low-density lipoprotein (LDL) receptors resulting in more efficient clearance of apolipoprotein B (ApoB)–containing lipoproteins [1, 2]. In short-term and long-term placebo- and ezetimibe-controlled phase 2 and 3 trials, evolocumab has been shown to significantly reduce LDL-cholesterol (LDL-C) and other atherogenic lipid fractions in participants with varying lipid phenotypes, cardiovascular risk, and baseline statin therapy [3–13]. In patients with mixed hyperlipidemia (characterized by elevated triglyceride and cholesterol levels), increased serum concentrations of remnant-like particles derived from either chylomicrons or very low–density lipoprotein (VLDL) are observed [14]. Clearance of remnant lipoproteins is complex and occurs through a variety of receptors, including the LDL-receptor [15]. While inhibition of PCSK9 with evolocumab has been shown to significantly reduce serum LDL-C, whether this effect would be similar in patients with higher circulating levels of triglycerides and remnant-like lipoproteins has not been evaluated.

In this analysis, we compared the efficacy and safety of evolocumab in participants from the phase 2 and 3 trials with mixed hyperlipidemia—baseline elevated LDL-C (≥2.0 mmol/L [75 mg/dL]) and elevated fasting triglycerides (≥1.7 mmol/L [150 mg/dL] to <4.5 mmol/L [400 mg/dL]) and those with only hypercholesterolemia—without elevated fasting triglyceride levels (<1.7 mmol/L). Additional comparison on the percentage of high-risk participants meeting LDL-C, non–high-density lipoprotein (HDL-C), and ApoB thresholds between the two groups was conducted.

## Methods

### Study Design

**Fig. 1:**
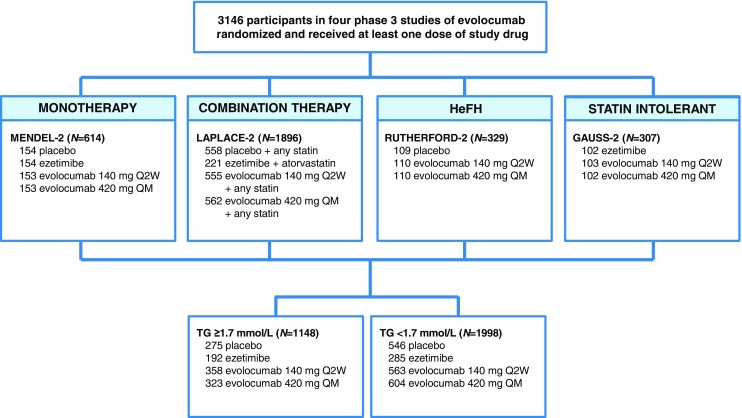
Participant disposition. *GAUSS* Goal Achievement After Utilizing an Anti-PCSK9 Antibody in Statin Intolerant Subjects, *HeFH* heterozygous familial hypercholesterolemia, *LAPLACE* LDL-C Assessment With PCSK9 Monoclonal Antibody Inhibition Combined With Statin Therapy, *MENDEL* Monoclonal Antibody Against PCSK9 to Reduce Elevated LDL-C in Subjects Currently Not Receiving Drug Therapy for Easing Lipid Levels, *Q2W* every 2 weeks, *QM* every month, *RUTHERFORD* Reduction of LDL-C With PCSK9 Inhibition in Heterozygous Familial Hypercholesterolemia Disorder, *TG* triglycerides

Efficacy was evaluated in four phase 3 randomized studies (*n* = 1148) [5, 9, 11, 12] and safety from the phase 2 and 3 studies (*n* = 2246) and their open-label extension studies (*n* = 1698) (Fig. 1) [3–13]. Efficacy analyses were based on 12-week phase 3 studies, while safety analyses included data from all available studies. Amgen sponsored and designed the trials and was responsible for data collection and analysis. Informed consent was obtained from each patient, and the study protocol conforms to the ethical guidelines of the Declaration of Helsinki as reflected in approval by the institution’s human research committee.

### Participants

Patients were eligible if they were adults aged 18 to 75 (phase 2 studies) or 18 to 80 (phase 3 studies) years with an LDL-C level of ≥2.0 mmol/L (75 mg/dL) and triglyceride level < 4.5 mmol/L (400 mg/dL). A fasting triglyceride level of ≥4.5 mmol/L (400 mg/dL) at screening was an exclusion criterion for these studies, but post-enrollment triglyceride levels may have exceeded 4.5 mmol/L. Full details of the exclusion criteria have been published elsewhere [16].

### Efficacy and Safety Endpoints

Efficacy analyses were based on 12-week phase 3 studies [5, 9, 11, 12]. Treatment differences were calculated vs. placebo and ezetimibe by pooling the data from evolocumab biweekly and monthly dosing groups. The co-primary endpoints were mean percentage change from baseline in LDL-C at weeks 10 and 12 and percentage change from baseline in LDL-C at week 12. Secondary endpoints included mean percentage changes in non–HDL-C, ApoB, HDL-C, and triglycerides. The mean percentage reduction from baseline in LDL-C at weeks 10 and 12 and percentage change from baseline in LDL-C at week 12 were not substantially different in the studies. The present analysis therefore reports mean percentage reduction from baseline in LDL-C, non–HDL-C, ApoB, and HDL-C at weeks 10 and 12. Safety analyses included data from all available studies.

### Statistical Analysis

The co-primary and co-secondary efficacy endpoints were analyzed using a repeated measures linear model, with terms for treatment group, study, the interaction of treatment and study, baseline LDL-C, dose frequency, visit, and the interaction of treatment with visit. The studies used for this analysis compared evolocumab vs. placebo, vs. ezetimibe, or vs. placebo or ezetimibe. Therefore, the analyses to assess the treatment effect of evolocumab vs. placebo only included studies that had a placebo treatment arm, and likewise for the comparison vs. ezetimibe. Cochran Mantel Haenszel tests or chi-squared tests were used for binary endpoints. Descriptive statistics were used to assess the incidence of adverse events and raised laboratory values. Statistical analysis was performed using SAS version 9.3 (SAS Institute, Cary, NC). Adverse events were coded using Medical Dictionary for Regulatory Activities version 17.0.

## Results

Baseline demographics, clinical characteristics, and lipids in patients with and without elevated triglycerides are shown in Table 1. Elevated triglyceride levels (≥1.7 mmol/L [150 mg/dL]) were more common in men, and there were significant differences by the participant’s race. This subgroup also had a greater prevalence of type 2 diabetes and multiple cardiovascular disease (CVD) risk factors, as well as increased levels of non–HDL-C and ApoB but lower HDL-C. Baseline mean (standard deviation) LDL-C was similar in patients with (3.4 [1.4] mmol/L) (129.9 mg/dL [52.4]) and without (3.3 [1.2] mmol/L) (127.6 [46.4]) elevated triglycerides. The proportions of participants on any statin treatment (72 % [*n* = 825] with elevated triglycerides, 73 % [*n* = 1450] without elevated triglycerides) and high-intensity statin treatment (32 % [*n* = 366], 33 % [*n* = 658]) were similar between participants with or without elevated triglycerides.

**Table 1:** Baseline demographics, disease characteristics, and lipid levels

Characteristic	TG ≥1.7 mmol/L at screening (*N* = 1148)	TG <1.7 mmol/L at screening (*N* = 1998)	*P*-value^a^
Age, mean (SD) (years)	57.4 (10.7)	58.0 (11.5)	NS
Female sex, *n* (%)	511 (44)	1042 (52)	<0.05
Race, *n* (%)			<0.05
White	1072 (93)	1806 (90)	
Asian	40 (4)	68 (3)	
Black or African American	20 (2)	104 (5)	
Other	16 (1)	20 (1)	
Coronary artery disease, *n* (%)	242 (21)	380 (19)	NS
Type 2 diabetes mellitus, *n* (%)	197 (17)	183 (9)	<0.05
≥2 cardiovascular risk factors, *n* (%)	560 (49)	610 (31)	<0.05
Metabolic syndrome without type 2 diabetes,^b^ *n* (%)	599 (52)	390 (20)	<0.05
LDL-C,^b^ mean (SD) (mmol/L)^c^	3.4 (1.4)	3.3 (1.2)	NS
TG, median (Q1, Q3) (mmol/L)	2.0 (1.6, 2.5)	1.1 (0.9, 1.4)	<0.05
HDL-C, mean (SD) (mmol/L)	1.2 (0.3)	1.5 (0.4)	<0.05
Non–HDL-C, mean (SD) (mmol/L)	4.4 (1.5)	3.9 (1.3)	<0.05
ApoB, mean (SD) (g/L)	1.1 (0.3)	1.0 (0.3)	<0.05
Statin treatment	825 (72)	1450 (73)	NS
High-intensity statin treatment	366 (32)	658 (33)	

*ApoB* apolipoprotein B, *HDL-C* high-density lipoprotein cholesterol, *LDL-C* low-density lipoprotein cholesterol, *NS* not significant, *Q* quartile, *SD* standard deviation, *TG* triglycerides

^a^Means were compared using t-tests. For TGs, medians were compared using a Wilcoxon test. Binary data was compared using a chi-squared test

^b^Metabolic syndrome is defined as having three or more of the following factors: elevated waist circumference (non-Asian: men ≥102 cm, women ≥88 cm; Asian: men ≥90 cm, women ≥80 cm), TG ≥1.7 mmol/L, low HDL-C (<1.0 mmol/L in men and <1.3 mmol/L in women), systolic blood pressure ≥ 130 mmHg or diastolic blood pressure ≥ 85 mmHg, or hypertension, or fasting glucose ≥100 mg/dL

^c^LDL-C was based on calculated values unless calculated LDL-C was <1.0 mmol/L or TG were >4.5 mmol/L, in which case the ultracentrifugation LDL-C value from the same blood sample was used instead, if available

### Efficacy Endpoints

**Fig. 2:**
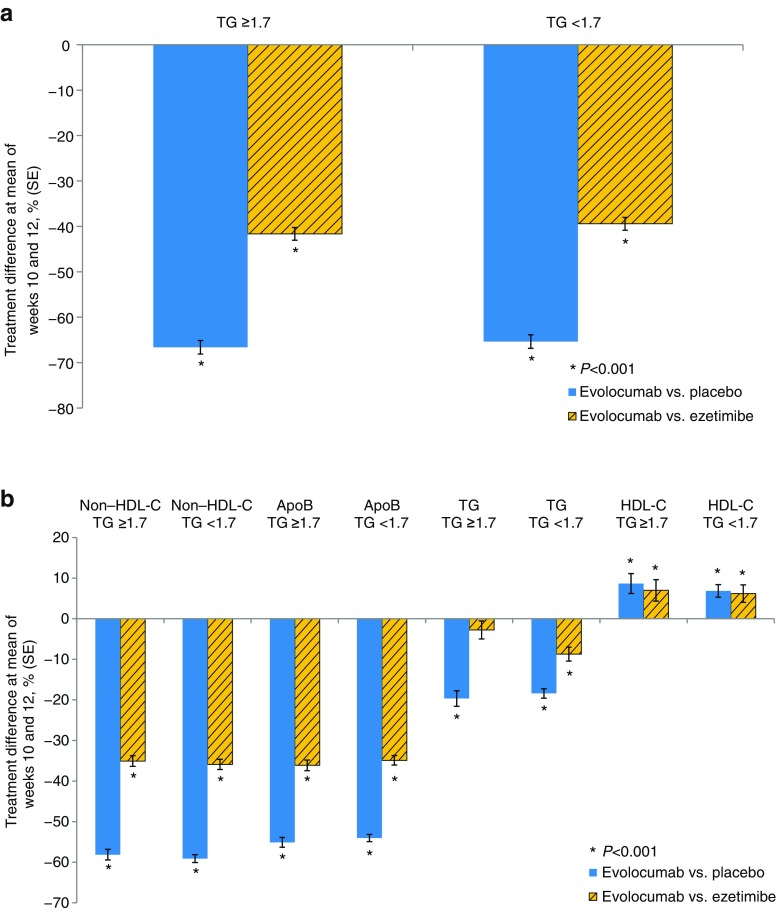
Effects of evolocumab vs. placebo or ezetimibe on (**a**) LDL-C levels and (**b**) other lipids in participants with or without elevated TG. LDL-C was based on calculated values unless calculated LDL-C was <1.0 mmol/L or TG were >4.5 mmol/L, in which case the ultracentrifugation LDL-C value from the same blood sample was used instead, if available. *ApoB* apolipoprotein B, *HDL-C* high-density lipoprotein cholesterol, *LDL-C* low-density lipoprotein cholesterol, *SE* standard error, *TG* triglycerides *P < 0.001

The treatment difference in mean percentage change from baseline to the mean of weeks 10 and 12 in LDL-C for evolocumab-treated participants with elevated triglycerides was approximately −67 % vs. placebo and −42 % vs. ezetimibe compared to −65 % vs. placebo and −39 % vs. ezetimibe in participants without elevated triglyceride levels (all *P* < 0.001) (Fig. 2a, Tables 2 and 3). Treatment differences for evolocumab vs. placebo and ezetimibe among those with or without elevated triglycerides also followed a similar pattern for non–HDL-C, ApoB, triglycerides, and HDL-C (Fig. 2b, Tables 2 and 3).

**Table 2:** LDL-C and other atherogenic lipids at baseline and at the mean of weeks 10 and 12 (ie, follow-up), participants with TG ≥1.7 mmol/L

	Placebo (*N* = 546)		Ezetimibe (*N* = 285)		Evolocumab (*N* = 1167)	
	Baseline	Follow-up	Baseline	Follow-up	Baseline	Follow-up
LDL-C^a^	3.17 (1.07)	3.23 (0.05)	3.50 (1.23)	2.83 (0.07)	3.34 (1.28)	1.37 (0.03)
TG	1.07 (0.84, 1.38)	1.11 (0.88, 1.48)	1.14 (0.88, 1.40)	1.10 (0.84, 1.38)	1.11 (0.87, 1.40)	0.96 (0.78, 1.24)
HDL-C	1.52 (0.47)	1.49 (0.02)	1.52 (0.43)	1.51 (0.02)	1.50 (0.44)	1.57 (0.01)
Non-HDL-C	3.70 (1.12)	3.79 (0.05)	4.05 (1.28)	3.36 (0.08)	3.88 (1.32)	1.81 (0.03)
ApoB^b^	0.93 (0.26)	0.94 (0.01)	0.99 (0.27)	0.86 (0.02)	0.96 (0.28)	0.49 (0.01)

Baseline values are mean (standard deviation) and the follow-up values are mean (standard error)—except for TG for which both baseline and follow-up values are median (interquartile range)

*ApoB* apolipoprotein B, *HDL-C* high-density lipoprotein cholesterol, *LDL-C* low-density lipoprotein cholesterol, *TG* triglycerides

^a^LDL-C was based on calculated values unless calculated LDL-C was <1.0 mmol/L or TG were >4.5 mmol/L, in which case the ultracentrifugation LDL-C value from the same blood sample was used instead, if available

^b^The number of patients with available data are, from left to right, 542, 531, 283, 277, 1161, and 1145

**Table 3:** LDL-C and other atherogenic lipids at baseline and at the mean of weeks 10 and 12 (ie, follow-up), participants with TG <1.7 mmol/L

	Placebo (*N* = 275)		Ezetimibe (*N* = 192)		Evolocumab (*N* = 681)	
	Baseline	Follow-up	Baseline	Follow-up	Baseline	Follow-up
LDL-C^a^	2.98 (1.08)	3.02 (0.08)	3.73 (1.45)	2.93 (0.09)	3.42 (1.40)	1.29 (0.03)
TG	1.92 (1.47, 2.41)	1.88 (1.45, 2.45)	2.12 (1.69, 2.71)	1.91 (1.35, 2.44)	1.99 (1.57, 2.54)	1.63 (1.28, 2.13)
HDL-C	1.23 (0.34)	1.20 (0.02)	1.18 (0.30)	1.18 (0.02)	1.22 (0.33)	1.30 (0.01)
Non-HDL-C	3.94 (1.24)	3.99 (0.08)	4.77 (1.62)	3.88 (0.11)	4.41 (1.52)	2.04 (0.04)
ApoB^b^	0.98 (0.27) [271]	1.00 (0.02) [271]	1.17 (0.36) [191]	0.99 (0.02) [187]	1.09 (0.34) [678]	0.54 (0.01) [663]

Baseline values are mean (standard deviation) and the follow-up values are mean (standard error)—except for TG for which both baseline and follow-up values are median (interquartile range)

*ApoB* apolipoprotein B, *HDL-C* high-density lipoprotein cholesterol, *LDL-C* low-density lipoprotein cholesterol, *TG* triglycerides

^a^LDL-C was based on calculated values unless calculated LDL-C was <1.0 mmol/L or TG were >4.5 mmol/L, in which case the ultracentrifugation LDL-C value from the same blood sample was used instead, if available

^b^The number of participants with available data are, from left to right, 271, 271, 191, 187, 678, and 663

**Fig. 3:**
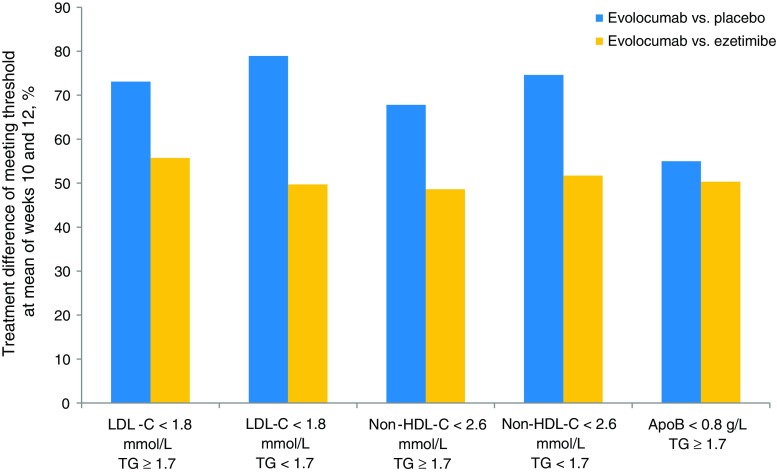
Treatment differences for meeting lipid and ApoB goals with evolocumab vs. placebo or ezetimibe in NCEP III–high-risk participants only with or without elevated TG. The numbers of participants represented were as follows: ≥1.7 mmol/L, 220 vs 93 (evolocumab vs placebo) and 131 vs 88 (evolocumab vs ezetimibe); <1.7 mmol/L, 323 vs 164 (evolocumab vs placebo) and 158 vs 65 (evolocumab vs ezetimibe). LDL-C was based on calculated values unless calculated LDL-C was <40 mg/dL or TG were >400 mg/dL, in which case the ultracentrifugation LDL-C value from the same blood sample was used instead, if available. *ApoB* apolipoprotein B, *LDL-C* low-density lipoprotein cholesterol, *NCEP* National Cholesterol Education Program, *HDL-C* high-density lipoprotein cholesterol, *TG* triglycerides

A greater proportion of participants with elevated triglycerides were classed as National Cholesterol Education Program (NCEP) high risk (41 %) compared with participants without elevated triglycerides (30 % NCEP high risk). We analyzed the proportion of NCEP III–high-risk participants meeting targets for LDL-C, non–HDL-C, and ApoB as proposed by several professional societies. A similarly high proportion of evolocumab-treated, NCEP III–high-risk patients with and without elevated triglycerides achieved the LDL-C target of <1.8 mmol/L (70 mg/dL) (82 % vs. 81 %, respectively) and <2.6 mmol/L (100 mg/dL) (92 % vs. 92 %, respectively). Significantly more participants without elevated triglycerides achieved the ApoB targets than participants with elevated triglycerides (*P* < 0.05). Additionally, significantly more participants without elevated triglycerides achieved the non–HDL-C target of <2.6 mmol/L (100 mg/dL) than participants with elevated triglycerides (85 % vs. 77 %, *P* < 0.05) (Table 4). Further breakdown of the treatment differences for meeting lipid and ApoB goals with evolocumab vs. placebo or ezetimibe with or without elevated triglycerides is shown in Fig. 3 (NCEP III–high-risk participants only).

**Table 4:** Percentage of NCEP–high-risk participants treated with evolocumab meeting lipid, non–HDL-C, and ApoB thresholds^a^

Goal^a^	TG ≥1.7 mmol/L, % (*n* = 284)	TG <1.7 mmol/L, % (*n* = 368)
LDL-C < 1.8 mmol/L^b^	82	81
LDL-C < 2.6 mmol/L^b^	92	92
Non–HDL-C < 2.6 mmol/L	77	85*
Non–HDL-C < 3.4 mmol/L	90	93
ApoB <0.8 g/L	85	93*
ApoB <0.9 g/L	90	94*

*ApoB* apolipoprotein B, *HDL-C* high-density lipoprotein cholesterol, *LDL-C* low-density lipoprotein cholesterol, *NCEP* National Cholesterol Education Program, *TG* triglycerides

^a^Thresholds met at mean of weeks 10 and 12

^b^LDL-C was based on calculated values unless calculated LDL-C was <1.0 mmol/L or TG were >4.5 mmol/L, in which case the ultracentrifugation LDL-C value from the same blood sample was used instead, if available

*P < 0.05, TG ≥1.7 mmol/L vs. TG <1.7 mmol/L based on chi-squared tests

### Safety Analyses

Evolocumab was generally well tolerated. Rates of adverse events were balanced between evolocumab vs. placebo or ezetimibe (Table 5).

**Table 5:** Safety in participants with or without elevated triglycerides

Category	Any placebo *n* (%)		Ezetimibe *n* (%)		Any evolocumab *n* (%)	
	TG ≥1.7 mmol/L (*N* = 592)	TG <1.7 mmol/L (*N* = 1136)	TG ≥1.7 mmol/L (*N* = 227)	TG <1.7 mmol/L (*N* = 327)	TG ≥1.7 mmol/L (*N* = 1427)	TG <1.7 mmol/L (*N* = 2721)
All AEs	282 (47.6)	574 (50.5)	124 (54.6)	155 (47.4)	699 (49.0)	1414 (52.0)
Grade ≥2^a^	147 (24.8)	290 (25.5)	64 (28.2)	57 (17.4)	317 (22.2)	630 (23.2)
Grade ≥3^a^	26 (4.4)	34 (3.0)	8 (3.5)	4 (1.2)	57 (4.0)	93 (3.4)
Grade ≥4^a^	4 (0.7)	3 (0.3)	0 (0.0)	0 (0.0)	7 (0.5)	17 (0.6)
Serious AEs	16 (2.7)	25 (2.2)	5 (2.2)	2 (0.6)	46 (3.2)	65 (2.4)
Leading to discontinuation of study drug	8 (1.4)	17 (1.5)	13 (5.7)	11 (3.4)	21 (1.5)	54 (2.0)
Serious	1 (0.2)	4 (0.4)	0 (0.0)	0 (0.0)	3 (0.2)	13 (0.5)
Non-serious	7 (1.2)	14 (1.2)	13 (5.7)	11 (3.4)	19 (1.3)	44 (1.6)
Fatal AEs	0 (0.0)	1 (0.1)	0 (0.0)	0 (0.0)	0 (0.0)	3 (0.1)
ALT or AST >3 × ULN	5 (0.9)	12 (1.1)	5 (2.2)	0 (0.0)	9 (0.6)	9 (0.3)
ALT or AST >5 × ULN	2 (0.3)	5 (0.4)	0 (0.0)	0 (0.0)	3 (0.2)	3 (0.1)
CK >5 × ULN	3 (0.5)	8 (0.7)	3 (1.3)	1 (0.3)	4 (0.3)	23 (0.9)
CK >10 × ULN	2 (0.3)	3 (0.3)	0 (0.0)	1 (0.3)	1 (0.1)	8 (0.3)

*AE* adverse event, *ALT* alanine aminotransferase, *AST* aspartate aminotransferase, *CK* creatine kinase, *TG* triglycerides, *ULN* upper limit of normal

^a^Graded according to Common Terminology Criteria for Adverse Events

## Discussion

This analysis evaluated the effects of evolocumab in participants with mixed hyperlipidemia (hypercholesterolemia with triglycerides ≥1.7 mmol/L [150 mg/dL]) and participants with hypercholesterolemia but without elevated triglycerides (<1.7 mmol/L [150 mg/dL]). Efficacy and safety of evolocumab treatment were similar in both groups.

The American Heart Association/American College of Cardiology guidelines recognize LDL as the major atherogenic lipoprotein and consequently identify LDL-C as the primary target of therapy [17]. However, triglyceride-rich particles (e.g., VLDL) also increase the risk of CVD, and the combination of high LDL-C coupled with high triglycerides represents a particularly atherogenic phenotype [18–20]. Consequently, professional societies have endorsed [18, 20, 21] non–HDL-C (LDL-C + VLDL-C) as the preferred target in patients with mixed hyperlipidemia [22]. Additional evidence supporting the contribution of other lipoproteins, beyond LDL, to increased cardiovascular risk includes an analysis of statin trials, which demonstrated that on-treatment levels of non–HDL-C are more strongly associated with future risk of atherosclerotic CVD events than LDL-C [23]. Also, in statin-treated subjects, some studies have shown that ApoB provides equivalent or superior discrimination of risk [24–28]. Furthermore, patients with an elevated triglyceride concentration have smaller LDL particles resulting in less efficient clearance via hepatic LDL receptors [29, 30]. This leads to higher LDL particle concentrations in patients with elevated triglycerides than would be predicted based on the level of LDL-C [29, 31]. Thus, several consensus documents propose a tiered approach for the assessment of treatment targets (LDL-C, non–HDL-C, and ApoB, or LDL particles) [32, 33].

Prior studies of evolocumab demonstrated significant LDL-C reductions of up to 75 % compared to placebo (in participants taking maximally tolerated statins), but its effect on patients with mixed hyperlipidemia was not formally evaluated. The results of this analysis demonstrate that cholesterol reduction with evolocumab is similar in patients with or without elevated triglycerides, with reductions of 67 % and 65 % vs. placebo, respectively. Similar to the reductions in LDL-C, evolocumab was equally efficacious in lowering non–HDL-C and ApoB in hypercholesterolemic participants regardless of whether the triglyceride level was elevated or not. Also shown is that 80 % to 90 % of participants achieved LDL-C, non–HDL-C, and ApoB thresholds (LDL-C < 1.8 mmol/L, non–HDL-C < 3.4 mmol/L, and ApoB <0.8 g/L targets), with the only exception in that 77 % of participants with elevated triglycerides achieved non–HDL-C < 2.6 mmol/L.

Strengths of our analysis include the broad group of participants studied including those from monotherapy, statin combination therapy, statin-intolerant and heterozygous familial hypercholesterolemia evolocumab trials as well as participants from placebo- and ezetimibe-controlled studies. Several limitations of the current study are also noted. One limitation is that we pooled data across randomized studies as a post-hoc analysis. Additionally, we did not analyze specimens for lipoprotein particle size and concentration in order to investigate the efficacy of evolocumab on the distribution of VLDL and LDL particles. Although we observed equivalent efficacy of evolocumab in participants with fasting triglycerides <4.52 mmol/L that are mainly transported in medium and small VLDL particles, none of the phase 2 or 3 studies included participants with baseline fasting triglycerides ≥4.52 mmol/L (400 mg/dL). Future studies would be useful to investigate the efficacy of evolocumab in patients with higher triglycerides that are transported in large VLDL particles (>4.5 mmol/L to <9.6 mmol/L) and chylomicrons.

## Conclusions

In participants with elevated triglycerides, evolocumab was well tolerated and resulted in statistically and clinically significant reductions of LDL-C, non–HDL-C, and ApoB levels vs. placebo and ezetimibe. Similar treatment effects were seen in participants without elevated triglycerides.

## Appendix: Clinical Trial Registration Identifiers

All studies used for this analysis were registered at ClinicalTrials.gov. The following lists display the identifiers for each study.

Phase 3, 12-week studies (efficacy and safety): NCT01763866, NCT01763827, NCT01763905, NCT01763918

Other phase 2 and 3 studies (safety): NCT01375751, NCT01375764, NCT01375777, NCT01380730, NCT01516879, NCT01763827, NCT01953328

Open-label extension studies (safety): NCT01849497, NCT01879319
